# Psychological well-being and its associated factors among university students in Sichuan, China

**DOI:** 10.3389/fpsyg.2025.1473871

**Published:** 2025-02-27

**Authors:** Juan Wan, Lei Hum Wee, Ching Sin Siau, Yin How Wong

**Affiliations:** 1 School of Software Engineering, Chengdu University of Information Technology, Chengdu, China; 2 Centre for Community Health Studies, Faculty of Health Sciences, Universiti Kebangsaan Malaysia, Kuala Lumpur, Malaysia; 3 School of Medicine, Faculty of Health and Medical Sciences, Taylor’s University, Subang Jaya, Selangor Darul Ehsan, Malaysia; 4 Digital Health and Medical Advancement Impact Lab, Taylor’s University, Subang Jaya, Selangor Darul Ehsan, Malaysia

**Keywords:** psychological well-being, positive psychology, university students, EPOCH, China

## Abstract

**Introduction:**

Psychological well-being refers to a mental state that allows an individual to achieve their full potential, be productive and innovative in their work, and manage everyday stressors. This study aims to assess university students’ psychological well-being by examining its associations with demographic factors such as gender, only child status, family background, grade level, financial aid status, and household income.

**Methods:**

This cross-sectional, online study utilized the EPOCH Measure of Adolescent Well-being to examine five positive well-being indicators: Engagement, Perseverance, Optimism, Connectedness and Happiness among Chinese university students aged 18–25 years (*N* = 4,911).

**Results:**

The results showed that females, only children, first-year students, and urban students exhibited higher levels of psychological well-being. Additionally, students who received the highest level of financial assistance were significantly associated with lower levels of psychological well-being.

**Conclusion:**

Identifying the influence of these socioeconomic factors on psychological well-being allows for targeted interventions to improve the mental health and social well-being of at-risk groups.

## 1 Introduction

Mental health, including emotional, psychological, and social well-being, is vital at every life stage (World Health Organization, 2022; Dattani et al., 2023; Mental Health, 2024). Well-being, as defined by Huppert (2009) and echoed by Diener et al. (2009), Ryan and Deci (2001), and Tov (2018), is the experience of health, happiness, and prosperity. It includes good mental health, high life satisfaction, a sense of purpose, and stress management (Davis, 2019), encompassing both hedonia (subjective well-being) and eudaimonia (psychological well-being). This aligns with Healthy People 2030’s definition of overall life satisfaction, reflecting both health and non-health factors (Office of Disease Prevention and Health Promotion, 2020).

Psychological well-being (PWB) is a multifaceted concept that encompasses aspects of positive functioning, self-realization, and the ability to face life’s challenges (Ryff, 1989). Various models have been proposed to explain this, such as Ryff (1989), Antonovsky (1979), Deci and Ryan (2008), and Seligman’s (2011) PERMA model of well-being in positive psychology. Seligman’s (2011) PERMA model in positive psychology suggests that psychological well-being is enhanced through fostering Positive emotions, Engagement, Relationships, Meaning, and Accomplishment. Kern et al. (2016) developed the EPOCH model, a framework for optimal adolescent functioning that aligns with Seligman’s PERMA model. It identifies five traits that support adult flourishing: Engagement, Perseverance, Optimism, Connectedness, and Happiness.

Emerging adulthood is crucial for students to gain autonomy from parents (Sussman and Arnett, 2014) and explore opportunities on campus, laying the foundation for full social integration post-graduation (Porru et al., 2022). The five factors of the EPOCH model are believed to support well-being in adulthood, aligning with the PERMA model’s key elements (Kern et al., 2016).

Studies across various countries, including the US, Australia, China, Iran, and Indonesia, support the cross-cultural invariance and equivalence of the EPOCH measure, though further validation and extension to diverse adolescent groups are needed (Kern et al., 2016; Kern et al., 2019; Setyandari, 2019; Taheri et al., 2021; Zeng and Kern, 2019). University students, as a distinct age group and social role in transition from adolescence to adulthood, face a myriad of unique challenges. Research conducted in Thailand has delved into the application of the EPOCH measure among college students (Rhein and Nanni, 2022).

University students have the potential to make significant societal contributions, and public awareness of their mental health issues is rising (Chen, 2024). However, anxiety, stress, depression, and challenging living circumstances remain significant barriers to students’ well-being. The reports of mental health issue among students have been increasing globally (Orygen, 2023; Youth Insight, 2023). According to the report from the Healthy Minds Network (HMS) for 2022–2023, 36% of surveyed students indicated receiving counseling or therapy. In China, the prevalence of anxiety, depression, sleep problems, and suicide attempts among college students has significantly increased over the past decade (Chen et al., 2022).

Studies suggest a global decline in student’ well-being at universities (Riva et al., 2020). Cross-cultural research examining happiness among college students from 24 countries reported that students from Southeast Asia (including China) had lower levels of happiness when compared to students from South America, sub-Saharan Africa, and the Caribbean (Peltzer et al., 2017). Additionally, research indicates that mental health tends to worsen after students enter college, with college students generally reporting worse mental health than non-students (Worsley et al., 2022). However, research focusing specifically on Chinese students’ well-being remains insufficient (Dobosz and Hetmańczyk, 2023).

There have been many one-child births in China since the enactment of the policy in 1980. As the birth rate declines, there will be a substantial number of families with only one child (National Bureau of Statistics of the People's Republic of China, 2023a). Non-only children face challenges in terms of having less access to material educational resources and parenting styles that are conducive to their skill development, compared to only children (Li et al., 2024). Previous studies have increasingly suggested that the well-being of rural children was affected by the impact of parental absence (Zhao and Yu, 2016), family economic status (Wen and Lin, 2012), and neighborhood environment (Zhao et al., 2017).

Financial stress is the cause of mental health deterioration, which results in poor academic performance, academic dishonesty, in addition to mental disorders such as anxiety, depression and Burnout Syndrome (Pokhrel et al., 2020). China’s student financial assistance system, funded by the government and supplemented by schools and society, supports needy students and rewards high achievers. Aid is provided in tiers to address varying needs through a well-structured mechanism (State Council of the People’s Republic of China, 2022). In this study, financial assistance is categorized into four classes: the first targeting specific vulnerable groups, the second for poorer families not in the first category, the third covering general poor families, and none for families receiving no assistance (State Council of the People’s Republic of China, 2018).

Addressing these challenges requires culturally tailored approaches to support students’ well-being, recognizing variations in psychological well-being across cultures. Studies suggest that while Western cultures often define well-being through positive affectivity, hedonic balance, and individualistic traits like autonomy and self-esteem (Joshanloo, 2014), Eastern cultures, including China, emphasize moral values, contentment, harmony, and spiritual fulfillment (Suh and Choi, 2018; Wirtz et al., 2009). This cultural distinction underscores the necessity of designing psychological well-being interventions that are both evidence-based and culturally appropriate. Demographic and socio-economic characteristics are important determinants of youth development and can be located in the exosystem (Gomez-Baya et al., 2023). The literature on the effects of socio-demographics on psychological well-being in university students has indicated differences by gender and cultural background, place of residence and neighborhood and family status (Zeng and Kern, 2019; Miles et al., 2020). China’s population size and geography present tremendous challenges and opportunities to improve the mental well-being of whole populations (Ni et al., 2020).

Kern et al. (2016) suggested five positive psychological characteristics in EPOCH model that support positive youth development. Moreover, most existing research was conducted with adolescent samples worldwide (Zeng and Kern, 2019; Taheri et al., 2021; Maurer et al., 2021; Setyandari, 2019; Rhein and Nanni, 2022), meaning that more research is needed with emerging adults in China. Addressing these issues in the Chinese context will ensure that all students have the resources and support they need to thrive both academically and personally. Thus, comprehending their level of psychological well-being and its associated factors is pivotal for fostering their mental health and holistic development.

In this study, we aimed to investigate the level of psychological well-being and its associations with demographic factors among Chinese undergraduate students, specifically gender, grade, poverty level, and locality. Within China’s unique socio-cultural and economic context, factors such as rural–urban divides, financial strain, and the legacy of the one-child policy are particularly relevant to understanding students’ well-being. Additionally, the disparities in educational resources and financial aid across regions further highlight the need to explore these demographic influences. This study seeks to provide culturally relevant insights to inform targeted interventions that promote the well-being of Chinese university students.

## 2 Materials and methods

### 2.1 Study design

Considering the time constraints and limited resources, we adopted a convenience sampling method to efficiently gather data from undergraduate students at our university between January and May 2024. The survey was conducted on the WJX online platform, leveraging students’ high digital engagement and aligning with current trends in online data collection (Lefever et al., 2007; Couper, 2017). Recruitment was facilitated through multiple official channels, including public elective courses, the university’s WeChat account (“Boya Xinyu”), counselor studio QQ groups, class meetings, and campus events. After providing informed consent online, participants completed the questionnaire anonymously on the platform. This approach ensured both the integrity of the data and the privacy of the participants, as no personally identifiable information was collected.

The sample size was large (*N* = 4,911) and represented diverse demographic and socioeconomic backgrounds, aligning closely with national data on gender distribution in higher education (National Bureau of Statistics of the People's Republic of China, 2023b). The rural–urban distribution also reflected broader trends, highlighting increased access to higher education for rural students despite national urban residency rates (National Bureau of Statistics of the People’s Republic of China, 2021). This large sample enhances the reliability and generalizability of the findings, capturing the demographic diversity of Chinese university students.

### 2.2 Instruments

The demographic information of the participants, including the year of study, gender, only child status, locality, financial aid status and domestic average income, was recorded.

The EPOCH Measure of Adolescent Well-being, available in both English (Kern et al., 2016) and Chinese (Zeng and Kern, 2019) was used in this study. It has previously demonstrated good internal consistency across all its subscales, with Cronbach’s *α* values ranging from 0.78 to 0.89. All participants responded to the 20-item EPOCH, which contains subscales of Engagement (e.g., ‘I get completely absorbed in what I am doing’), Perseverance (e.g., ‘I finish whatever I begin’), Optimism (e.g., ‘I think good things are going to happen to me’), Connectedness (e.g., ‘When I have a problem, I have someone who will be there for me’) and Happiness (e.g., ‘I love life’). Each item is rated on a 5-point Likert scale ranging from 1 ‘Almost never’ to 5 ‘Almost always’, reflecting how frequently the participants experience for each aspect of well-being. These dimensions collectively provide a comprehensive assessment of positive psychological functioning and well-being in adolescents.

### 2.3 Participants

Data were collected from university students at Chengdu University of Information Technology, with 4,911 out of a total of 25,000 students (19.6%). The inclusion criteria for participants in this study are Chinese nationality, university students aged between 18 and 25 years, and full-time enrollment at the university during the study. Participants were excluded if they were part-time students, international students, or not enrolled as students, as they either had a relatively small presence at the university or were unable to commit to the entire duration of the survey.

Ethical considerations were addressed by ensuring anonymity, avoiding identifiable data, and obtaining electronic informed consent with clear communication of participants’ rights. All students completed the questionnaire anonymously, ensuring data integrity and respect for privacy.

### 2.4 Data analysis

Descriptive statistics were used to compute means, standard deviations, frequencies, and percentages. Independent sample t-tests and analysis of variance were employed to assess the statistical significance of differences between groups, and multiple linear regression analysis was used to explore the complex interactions between variables. IBM SPSS for Mac (version 26.0) was used to analyze the data.

## 3 Results

### 3.1 Description of the EPOCH scale

Compared with previous studies, the Cronbach’s alpha value obtained in this study is relatively high (Cronbach’s *α* = 0.952). Specifically, in previous studies, the Indonesian version (Simanjuntak et al., 2023) had a Cronbach’s alpha value of 0.889, and the Swedish version (Maurer et al., 2021) had a Cronbach’s alpha value of 0.91. The subscales had acceptable reliability: Engagement (*α* = 0.873), Perseverance (*α* = 0.822), Optimism (*α* = 0.838), Connectedness (*α* = 0.846), and the strongest one was Happiness (*α* = 0.917).

**Figure 1:**
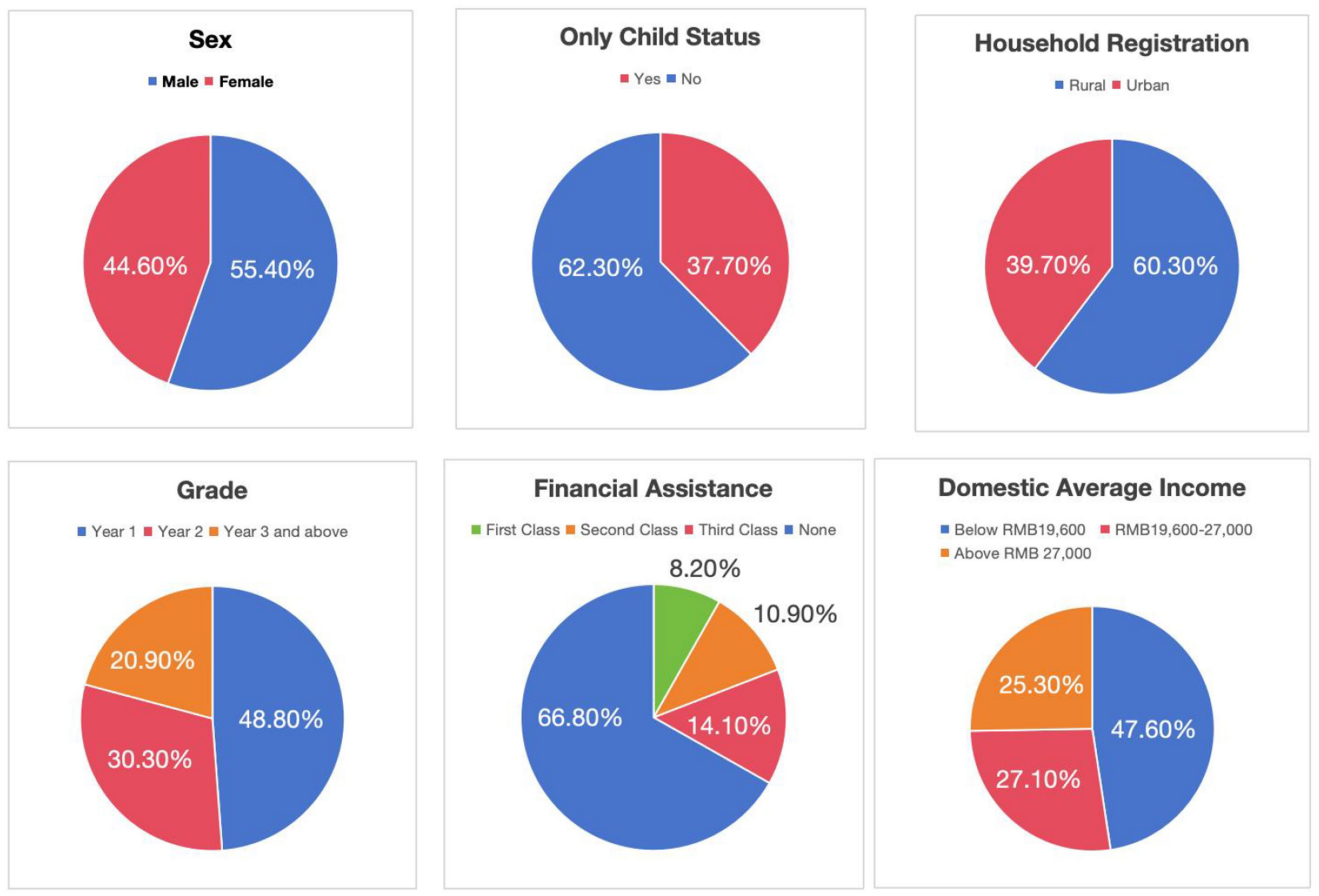
Demographic characteristics of the participants (*N* = 4,911).

In the research sample, males accounted for more than half, reaching 55.40% (*n* = 2,721). The proportion of only children was less than half, at 37.70% (*n* = 1849). Over 60% of the students came from rural areas, accounting for 60.30% (*n* = 2,963) of the total. Nearly half of the students were first-year students, accounting for 48.80% (*n* = 2,398), while students in the third year or higher accounted for less than one-fifth, at 20.90% (*n* = 1,024). Only 8.20% (*n* = 404) of students received first-class assistance, while more than 60%, specifically 66.80% (*n* = 3,281), did not receive any financial assistance at all. Nearly half of the students were from lower-income families, representing 47.60% (*n* = 2,338) (see Figure 1).

The study found significant differences between males and females, with males (3.32 ± 0.76) scoring significantly lower than females (3.47 ± 0.70) on the EPOCH total score. Only children (3.45 ± 0.76) had significantly higher EPOCH total score compared to non-only children (3.35 ± 0.73), and this trend was consistent across all five dimensions. Additionally, urban students (3.47 ± 0.74) scored significantly higher than rural students (3.33 ± 0.73) on the overall EPOCH dimension.

First-year students had the highest EPOCH total score (3.50 ± 0.73) compared to other academic years. Students receiving higher levels of financial assistance exhibited poorer performance on various psychological indicators, although not all differences were statistically significant. Those receiving financial assistance had significantly lower scores in Happiness (3.29 ± 0.95) and overall EPOCH total score (3.29 ± 0.70) compared to students without financial assistance. Students from lower-income families had significantly lower scores in overall EPOCH total score (3.29 ± 0.76) compared to other groups (see Table 1).

**Table 1:** Score of different domains in EPOCH scale according to demographic groups.

Variable	Total	Engagement	Perseverance	Optimism	Connectedness	Happiness	EPOCH (total)
	Frequency (*n*), Percentage (%)	Mean (SD)	Mean (SD)	Mean (SD)	Mean (SD)	Mean (SD)	Mean (SD)
Sex							
Male	2721 (55.40)	3.30 (0.84)	3.24 (0.82)	3.26 (0.90)	3.45 (0.86)	3.34 (0.92)	3.32 (0.76)
Female	2190 (44.60)	3.32 (0.79)	3.24 (0.80)	3.40 (0.87)	3.84 (0.82)	3.55 (0.86)	3.47 (0.70)
P-value	-	0.657	0.941	< 0.001	< 0.001	< 0.001	< 0.001
Only child status							
Yes	1849 (37.70)	3.36 (0.85)	3.29 (0.83)	3.40 (0.91)	3.71 (0.87)	3.49 (0.91)	3.45 (0.76)
No	3062 (62.30)	3.28 (0.80)	3.21 (0.79)	3.28 (0.88)	3.57 (0.86)	3.39 (0.89)	3.35 (0.73)
*P*-value	-	< 0.001	< 0.001	< 0.001	< 0.001	< 0.001	< 0.001
Household registration							
Rural	2963 (60.30)	3.27 (0.81)	3.20 (0.79)	3.26 (0.88)	3.55 (0.86)	3.38 (0.88)	3.33 (0.73)
Urban	1948 (39.70)	3.37 (0.83)	3.29 (0.83)	3.42 (0.90)	3.74 (0.86)	3.51 (0.92)	3.47 (0.74)
*P*-value	-	< 0.001	< 0.001	< 0.001	< 0.001	< 0.001	< 0.001
Grade							
Year 1	2398 (48.80)	3.40 (0.83)	3.32 (0.80)	3.46 (0.88)	3.73 (0.86)	3.58 (0.89)	3.50 (0.73)
Year 2	1489 (30.30)	3.18 (0.81)	3.12 (0.80)	3.16 (0.89)	3.47 (0.87)	3.26 (0.88)	3.23 (0.74)
Year 3 and above	1024 (20.90)	3.27 (0.80)	3.25 (0.81)	3.25 (0.87)	3.61 (0.83)	3.34 (0.91)	3.34 (0.71)
*P*-value	-	< 0.001	< 0.001	< 0.001	< 0.001	< 0.001	< 0.001
Financial assistance							
First class	404 (8.20)	3.23 (0.87)	3.21 (0.84)	3.22 (0.95)	3.53 (0.86)	3.29 (0.95)	3.29 (0.79)
Second class	536 (10.90)	3.29 (0.81)	3.23 (0.78)	3.28 (0.81)	3.52 (0.81)	3.37 (0.85)	3.34 (0.70)
Third class	690 (14.10)	3.26 (0.82)	3.18 (0.79)	3.27 (0.89)	3.56 (0.87)	3.36 (0.90)	3.33 (0.74)
None	3281 (66.80)	3.33 (0.82)	3.25 (0.81)	3.35 (0.89)	3.67 (0.87)	3.47 (0.90)	3.42 (0.74)
*P*-value	-	0.015	0.161	0.004	< 0.001	< 0.001	< 0.001
Domestic average income							
Below RMB19,600	2338 (47.60)	3.24 (0.83)	3.16 (0.81)	3.23 (0.90)	3.49 (0.88)	3.33 (0.92)	3.29 (0.76)
RMB19,600-27,000	1332 (27.10)	3.29 (0.78)	3.23 (0.76)	3.33 (0.85)	3.63 (0.82)	3.43 (0.85)	3.38 (0.69)
Above RMB 27,000	1241 (25.30)	3.46 (0.83)	3.39 (0.82)	3.50 (0.89)	3.88 (0.82)	3.62 (0.89)	3.38(0.72)
*P*-value	-	< 0.001	< 0.001	< 0.001	< 0.001	< 0.001	< 0.001

SD, Standard deviation; First class targets specific vulnerable groups, such as families with registered cards, disabled families, families receiving subsistence allowances, and orphans; Second class is for poorer families that do not fall into the above four categories; Third class covers general poor families; None represents families that do not receive any financial assistance.

### 3.2 Multiple linear regression analysis using the EPOCH scale across different groups

Family economic status was measured using the financial assistance category, as it reflects institutional assessments of students’ financial circumstances based on standardized criteria. This approach avoided multicollinearity with domestic average income, ensuring model clarity and stability in the regression analysis.

The results of the multiple regression analysis identified several significant predictors of psychological well-being. Domestic average income was not entered into the model due to multicollinearity with financial assistance. Only child status was positively associated with higher scores in the Connectedness dimension (*B* = 0.10, 95% *CI* [0.05, 0.15], *p* < 0.001). Additionally, being a first-year student was positively correlated with higher levels of psychological well-being (*B* = 0.10, 95% *CI* [0.11, 0.21], *p* < 0.001). Conversely, males were found to have lower levels of psychological well-being (*B* = −0.14, 95% *CI* [−0.18, −0.10], *p* < 0.001). Similarly, students from rural areas ( *B* = −0.09, 95% *CI* [−0.13, −0.04], *p* < 0.001) and students receiving first-class financial assistance ( *B* = −0.08, 95% *CI* [−0.16, −0.01], *p* < 0.01) also demonstrated lower levels of psychological well-being (see Table 2).

**Table 2:** Multiple linear regression analysis using the EPOCH scale across different groups.

Variable	Engagement		Perseverance		Optimism		Connectedness		Happiness		EPOCH (total)	
	*B*	95% *CI*	*B*	95% *CI*	*B*	95% *CI*	*B*	95% *CI*	*B*	95% *CI*	*B*	95% *CI*
Sex												
Male	-	-	-	-	−0.13***	−0.18, −0.08	−0.38***	−0.43, −0.33	−0.20***	−0.25, −0.15	−0.14***	−0.18, −0.10
Female†	-	-	-	-	0.000		0.000		0.000		0.000	
Only child status												
Yes	0.06*	0.01, 0.11	0.07*	0.02, 0.12	0.08**	0.03, 0.14	0.10***	0.05, 0.15	0.08**	0.02, 0.13	0.08**	0.03, 0.12
No†	0.000	-	0.000	-	0.000	-	0.000	-	0.000	-	0.000	-
Household registration												
Rural	−0.06*	−0.12, −0.01	−0.07**	−0.12, −0.02	−0.13***	−0.18, −0.07	−0.11***	−0.16, −0.06	−0.07*	−0.13, −0.01	−0.09***	−0.13, −0.04
Urban†	0.000	-	0.000	-	0.000	-	0.000	-	0.000	-	0.000	-
Grade												
Year 1	0.15***	0.09, 0.21	0.08*	0.02, 0.13	0.22***	0.16, 0.28	0.12***	0.06, 0.18	0.24***	0.18, 0.31	0.10***	0.11, 0.21
Year 2	−0.08*	−0.14, −0.01	−0.15***	−0.22, −0.09	−0.07*	−0.14, 0.00	−0.12***	−0.19, −0.05	−0.07	−0.14, 0.00	−0.10**	−0.16, −0.04
Year 3 and above†	0.000	-	0.000	-	0.000	-	0.000	-	0.000	-	0.000	-
Financial assistance												
First class	−0.08	−0.16, 0.01	-	-	−0.08	−0.17, 0.02	−0.09*	−0.18, −0.01	−0.04**	−0.24, −0.05	−0.08*	−0.16, −0.01
Second class	−0.02	−0.09, 0.06	-	-	−0.03	−0.11, 0.05	−0.12**	−0.20, −0.04	−0.08	−0.16, 0.01	−0.05	−0.12, 0.02
Third class	−0.05	−0.12, 0.02	-	-	−0.04	−0.12, 0.03	−0.07	−0.14, 0.00	−0.08*	−0.15, 0.00	−0.06	−0.12, 0.01
None†	0.000	-	-	-	0.000	-	0.000	-	0.000	-	0.000	-

^†^Reference Group; * < 0.05, ** < 0.01, *** < 0.001 (Significant *p*-values); 95% *CI*, 95% Confidence Interval; *B*, Regression coefficient.

## 4 Discussion

In this study, we explored the psychological well-being of Chinese university students by analyzing the correlation between the EPOCH Measure of Adolescent Well-being and their demographic characteristics. We found that being female, an only child, an urban student, and in the first grade predicted higher overall well-being among university students.

The results indicate that male students had significantly lower total EPOCH scores, which corresponds to previous research findings that girls in China reported higher psychological well-being than boys (Chen et al., 2009; Liu and Zhao, 2016; Wong et al., 2009; Geng and He, 2022). Both Western and Eastern scholars have noted that Confucianism, a prevalent cultural heritage in East Asia, regards men as the mainstay of social order and family, while women are primarily viewed as caregivers and mothers (Hamilton, 1990; Qing, 2020).

This aspect of traditional Chinese culture influences societal values, shaping parental expectations that male students should be future providers and achieve career success, thereby creating significant pressure and restricting their emotional expression despite experiencing emotions as intensely as women (Heesacker et al., 1999; Kiselica, 2005; Wester et al., 2002). This could affect their ability to express emotions and establish supportive relationships, which may further impact their scores in terms of psychological well-being.

Ryff and colleagues (Ryff and Keyes, 1995; Ahrens and Ryff, 2006; Karasawa et al., 2011) consistently found that women score higher than men in positive relations with others across both Eastern and Western cultures. Women also tend to report greater emotional reactivity and fusion with others (Peleg, 2022), whereas men report higher emotional cutoff and are expected to appear confident and assertive, often suppressing vulnerable emotions (Mahalik et al., 2003; Vandello and Bosson, 2013; Peleg and Peleg, 2023). These tendencies contribute to higher scores in Connectedness and Optimism among female students.

Although research indicates that female students on average experience higher levels of academic stress (Graves et al., 2021; Devchoudhury and Devasagayam, 2022), it also reveals that they are happier at school than males (Pramod, 1996; Bhansali and Trivedi, 2008; Toraman et al., 2022), which is consistent with the findings of this study.

Women were more likely to use adaptive coping strategies (Tamres et al., 2002), whereas men were more likely to use maladaptive and avoidance coping strategies (Gentry et al., 2007). Empirical evidence consistently indicates a positive link between greater social support and improved mental health outcomes (Özer et al., 2021; McLean et al., 2023). This may have led to female students exhibited higher scores in Connectedness, Optimism, and Happiness, ultimately resulting in higher EPOCH total scores.

Past research on gender differences in Engagement has yielded mixed results. Some studies suggest women exhibit stronger academic Engagement (Zusman et al., 2005; Sax, 2008), possibly varying by field of study (Mastekaasa and Smeby, 2008). Others find males more polarized in Engagement levels (Hu and Kuh, 2002) or no consistent gender pattern (Zhao et al., 2005). Kuh (2003) observed higher female Engagement, while EPOCH research noted higher male Engagement during adolescence (Kern et al., 2016). These inconsistencies likely stem from varying definitions and measurements of Engagement (Hu and Kuh, 2002; Kuh, 2003; Mastekaasa and Smeby, 2008). In our study, no significant gender differences in Engagement were found, supporting its conceptualization as a universal psychological capacity. Further research is needed to explore these findings.

Our research revealed no significant sex difference in Perseverance, aligning with previous findings (Ryan, and andBeamish, P., 2018). Boys tend to show grit directly, whereas girls exhibit Perseverance indirectly through self-control. This suggests gender differences may be mediated by the expression of Perseverance. Perseverance is more linked to internal motivations than external factors, reducing gender gaps (Zeng and Kern, 2019). As Perseverance is closely tied to school Engagement and self-control, which develop similarly in both sexes, this explains the lack of significant differences. Thus, boys and girls may show similar Perseverance levels when considering internal motivations and self-regulation.

This sex difference indicates that nuanced variations in well-being scores may be influenced by complex, interacting social, cultural, and individual factors, and understanding these differences can help tailor interventions and support.

The results also found that only children reported higher scores across all dimensions, with a significantly higher total EPOCH score. The implementation of the one-child policy led most Chinese families to prioritize investing in their only child, particularly in their education (Lee, 2012; Li, 2001; Zhang and Goza, 2006). Only children receive high levels of parental attention and concentrated family resources, along with more patience and support, which enhances their prosocial behavior (Carlo et al., 2011; Li et al., 2024), thereby enhancing their sense of well-being. Research by Wolke and Skew (2012) suggests that only children may be happier, as they avoid sibling competition and conflicts, thereby reducing psychological pressure (Wolke and Skew, 2012).

This study found that urban students scored significantly higher on psychological well-being compared to rural students. Although there is evidence of significant cross-country and cross-regional variation in rural–urban differences in subjective well-being (Easterlin et al., 2011; Wang and Wang, 2016; Navarro et al., 2020), numerous studies have shown that urban populations generally report higher subjective well-being due to factors like better economic prospects and higher living standards (Easterlin et al., 2011; Wang and Wang, 2016; Navarro et al., 2020; Viganó et al., 2019; Burger et al., 2020). Tripathy and Sahu (2021) further supports this, finding urban students have better mental health compared to rural counterparts, who often lack basic amenities and face frustration from limited social opportunities (Dasinger and Gibson, 2024).

In China, rural students face unique challenges, including heavy family economic burdens and limited parental support due to low education levels and insufficient emotional care (Chen et al., 2020; Pillay et al., 2020; Wu et al., 2024). These factors, combined with fewer educational resources and social support, hinder their academic and psychological development (Li and Ranieri, 2013; Cui et al., 2021; Chow et al., 2019; Yao et al., 2014). The transition to urban universities exacerbates these challenges, creating a cultural gap as rural students struggle with unfamiliar norms, anxiety, and feelings of inadequacy (Sennett, 1973). Financial constraints further isolate them, limiting participation in social events and additional learning opportunities (Li, 2017). Surrounded by wealthier urban peers, rural students are reminded of their social and economic disadvantages, reinforcing exclusion and incompetence (Bourdieu, 1984). These combined factors contribute to the lower well-being reported by rural students compared to their urban counterparts. Based on the findings, universities in China could implement practical interventions such as providing targeted mental health support and financial aid to rural students, along with offering cultural adaptation programs and enhancing social integration opportunities to bridge the rural–urban gap.

A few studies have identified that the significant differences are primarily attributable to the combined effects of resource disparities, social environments and networks (Bosworth and Schaie, 1997; Gaspar et al., 2023), cultural differences (Suh and Oishi, 2002), and psychological stress (Hudd et al., 2000). To improve the mental health of rural students, it is essential to foster collaborative efforts from the government, educational institutions, and various societal sectors (Garbacz et al., 2022).

The study revealed that first-year students reported the highest level of psychological well-being, while second-year students exhibited the lowest. This aligns with previous research indicating that first-year students benefit from greater institutional support, social activities, and the excitement of transitioning into university life, contributing to their elevated well-being (Ahuja and Garg, 2021).

Second-year students face unique and significant challenges that contribute to their decreased well-being. Bewick et al. (2010) observed heightened anxiety among them due to increased academic, developmental, and social demands, coupled with reduced institutional support. This transition period, marked by identity formation and goal re-evaluation (Sterling, 2018), is a critical adjustment phase. Schreiner (2007) termed it a “developmental crisis,” highlighting sophomores’ uncertainty about their future direction and purpose, exacerbated by academic pressures and the need to choose a major (Schreiner and Pattengale, 2000). Other studies support our findings and suggest that among second-year students, a decrease in perceived competence due to first-year demands leads to lower happiness, while higher stress is attributed to increased studying, time management difficulties, and less established social support and coping mechanisms (Shek et al., 2017; Allen and Hiebert, 1991; Misra and McKean, 2000; Liu et al., 2019). These findings highlight the need for targeted, year-specific interventions to address the academic, developmental, and emotional challenges faced by second-year students, supporting their well-being and progression through university.

Students receiving higher levels of financial assistance performed worse on psychological indicators, particularly those receiving first-class financial assistance. This may suggest that while financial aid may alleviate some economic pressures, it alone may not be sufficient to fully compensate for all psychological deficiencies among students. Receiving financial assistance may indicate a lower socioeconomic status. A systematic review revealed that higher socioeconomic status was associated with greater subjective well-being (Tan et al., 2020). Future research could delve deeper into how financial assistance can be made more effective in fostering psychological development among students and how potential adverse effects could be mitigated.

Understanding these factors contributes to the development of targeted strategies and interventions to support the psychological well-being of Chinese university students. While these predictors have been identified in previous studies, our research offers a comprehensive analysis within a specific context, highlighting their combined influence and emphasizing the need for tailored approaches. For instance, gender psychological well-being programs can address societal pressures on male students’ emotional expression. Enhanced collaboration between parents and universities can benefit non-only children, leveraging family resources to boost well-being.

Moreover, our study underscores the criticality of addressing the urban–rural divide by proposing increased investment in rural educational resources and mental health services. We suggest concrete measures, such as establishing scholarships, offering internships, and organizing mental health outreach activities, to support rural students. Additionally, we propose implementing stress management workshops and academic counseling services to alleviate academic stress for second-year students, which is a unique contribution to practical intervention strategies.

We acknowledge the limitations of this study, including its focus on a single university and the use of convenience sampling, which may affect the generalizability of the findings. Future research should address these limitations by incorporating data from multiple universities, involving more diverse and representative populations, and employing randomized sampling methods. Such approaches will help ensure greater external validity and provide a more comprehensive understanding of psychological well-being among university students in China.

## Data Availability

The raw data supporting the conclusions of this article will be made available by the authors, without undue reservation.
