# Correction: Psychological well-being and its associated factors among university students in Sichuan, China

**DOI:** 10.3389/fpsyg.2025.1765564

**Published:** 2026-01-09

**Authors:** Juan Wan, Lei Hum Wee, Ching Sin Siau, Yin How Wong

**Affiliations:** 1 School of Software Engineering, Chengdu University of Information Technology, Chengdu, China; 2 Centre for Community Health Studies, Faculty of Health Sciences, Universiti Kebangsaan Malaysia, Kuala Lumpur, Malaysia; 3 School of Medicine, Faculty of Health and Medical Sciences, Taylor's University, Subang Jaya, Selangor Darul Ehsan, Malaysia; 4 Digital Health and Medical Advancement Impact Lab, Taylor's University, Subang Jaya, Selangor Darul Ehsan, Malaysia

**Keywords:** psychological well-being, positive psychology, university students, EPOCH, China

Author Lei Hum Wee was erroneously assigned to affiliation Centre for Community Health Studies, Faculty of Health Sciences, Universiti Kebangsaan Malaysia, Kuala Lumpur, Malaysia. This affiliation has now been removed for author Lei Hum Wee.

The original version of this article has been updated.

